# Multi-Parametric Evaluation of Chronic Kidney Disease by MRI: A Preliminary Cross-Sectional Study

**DOI:** 10.1371/journal.pone.0139661

**Published:** 2015-10-02

**Authors:** Pottumarthi V. Prasad, Jon Thacker, Lu-Ping Li, Muhammad Haque, Wei Li, Heather Koenigs, Ying Zhou, Stuart M. Sprague

**Affiliations:** 1 Department of Radiology, NorthShore University HealthSystem, Evanston, Illinois, United States of America; 2 Department of Biomedical Engineering, Northwestern University, Evanston, Illinois, United States of America; 3 Center for Biomedical Research Informatics, NorthShore University HealthSystem, Evanston, Illinois, United States of America; 4 Department of Medicine, NorthShore University HealthSystem, Evanston, Illinois, United States of America; University Medical Center Utrecht, NETHERLANDS

## Abstract

**Background:**

The current clinical classification of chronic kidney disease (CKD) is not perfect and may be overestimating both the prevalence and the risk for progressive disease. Novel markers are being sought to identify those at risk of progression. This preliminary study evaluates the feasibility of magnetic resonance imaging based markers to identify early changes in CKD.

**Methods:**

Fifty-nine subjects (22 healthy, 7 anemics with no renal disease, 30 subjects with CKD) participated. Data using 3D volume imaging, blood oxygenation level dependent (BOLD) and Diffusion MRI was acquired. BOLD MRI acquisition was repeated after 20 mg of *iv* furosemide.

**Results:**

Compared to healthy subjects, those with CKD have lower renal parenchymal volumes (329.6±66.4 *vs*. 257.1±87.0 ml, p<0.005), higher cortical R2* values (19.7±3.2 *vs*. 23.2±6.3 s^−1^, p = 0.013) (suggesting higher levels of hypoxia) and lower response to furosemide on medullary R2* (6.9±3.3 *vs*. 3.1±7.5 s^−1^, p = 0.02). All three parameters showed significant correlation with estimated glomerular filtration rate (eGFR). When the groups were matched for age and sex, cortical R2* and kidney volume still showed significant differences between CKD and healthy controls. The most interesting observation is that a small number of subjects (8 of 29) contributed to the increase in mean value observed in CKD. The difference in cortical R2* between these subjects compared to the rest were highly significant and had a large effect size (Cohen’s d = 3.5). While highly suggestive, future studies may be necessary to verify if such higher levels of hypoxia are indicative of progressive disease. Diffusion MRI showed no differences between CKD and healthy controls.

**Conclusions:**

These data demonstrate that BOLD MRI can be used to identify enhanced hypoxia associated with CKD and the preliminary observations are consistent with the chronic hypoxia model for disease progression in CKD. Longitudinal studies are warranted to further verify these findings and assess their predictive value.

## Introduction

Chronic kidney disease (CKD) is a significant public health problem, both in the United States [1] and other parts of the world [2]. Staging of CKD was proposed by National Kidney Foundation in 2002 primarily based on estimations of glomerular filtration rate (GFR) [1] in order to stratify patients at different stages of kidney disease. Using this definition of CKD, the analysis of the Third National Health and Nutrition Examination Survey (NHANES III) estimated that CKD is a common medical problem, affecting over 26 million people in the US. However, only about 5% percent of these will progress to end stage kidney disease (ESKD) where the only therapeutic options are dialysis or kidney transplantation. The care of patients with ESKD is expensive and it is estimated that Medicare spends over $20 billion annually accounting for almost 7% of its budget [3]. A key unmet clinical need is the ability to identify subjects with progressive CKD so that they can be aggressively managed to prevent or slow disease progression and hence minimize the number reaching ESKD. In other words, which 5% of the 26 million will actually progress to ESKD? This has led to interest in the development of alternate biomarkers [4] and/or combination of markers [5] that may identify subjects at risk of progression. Identifying subjects at risk of progression could provide a window of opportunity to intervene as well as facilitate effective clinical trials because of the ability to enrich the study population [6].

Irrespective of the initial causes, progressive CKD often results in widespread tissue scarring that leads to complete destruction of kidney parenchyma and ESKD. It is believed that renal fibrosis provides the best predictive indicator of progression to ESKD [7]. Currently, biopsy is the only viable option to evaluate fibrosis and so no data exists regarding presence of fibrosis at early stages of progression. The progressive scarring is now becoming accepted to be related to chronic ischemia created by the loss of peritubular capillaries [7]. Data in animal models provide a compelling argument for hypoxia as a primary mediator of progressive scarring in the kidney [7]. But the relevance to humans is not yet clear. Recent and limited data based on the expression of hypoxia inducible factor (HIF) in biopsy samples suggest presence of hypoxia in clinical settings [8,9]. Dependence on biopsy samples makes this approach not viable for more widespread utility, especially in patients with early stages of progression. BOLD MRI offers the only known non-invasive option to evaluate renal hypoxia. The primary objective in undertaking the current study was to examine whether CKD was associated with increased levels of hypoxia as evaluated by BOLD MRI. Since diffusion MRI has been shown to be sensitive to the presence of fibrosis [10,11] and kidney size has been shown to change in CKD [12], we also evaluated apparent diffusion coefficient (ADC) and renal volume measurements by MRI. These three MRI markers were evaluated in a cross-sectional study with patients at different stages of CKD. Healthy subjects with no known renal disease were used as controls. Further, to evaluate potential contribution of reduced hematocrit on BOLD MRI measurements, subjects with anemia due to non-renal causes were also included.

## Methods

### Subjects

All procedures were performed with approval from the institutional review board of the NorthShore University HealthSystem Research Institute and written subject consent. Fifty nine subjects participated. Table 1 summarizes the demographic, clinical and laboratory findings for the different groups. Subjects were instructed to fast overnight before coming for the MRI scans performed in the morning. They were also asked to refrain from using non-steroidal anti-inflammatory drugs (NSAIDs) for a week before the scheduled MRI scans.

**Table 1:** Summary of clinical & laboratory data for different groups.

		Healthy	Anemic	CKD
# of cases		22	7	30
	Male	15	0	15
	Female	7	7	15
Age (yrs)		50.4±15.3	44.5±10.3	61.6±10.4
Laboratory findings	eGFR (ml/min per 1.73 m^2^) / CKD stage	91.7±12.8	90.3±12.4	Stage 2 (n = 10); 3 (n = 10); 4 (n = 7); 5 (n = 3)
	Hb (g/dl)	—	11.1±0.69	12.6±1.8
	Urinary protein (mg/mg UCr)	—	—	0.83±0.9
	Systolic Blood pressure (mm Hg)	—	122.2±.34.0	130.7±25.6
	Diastolic Blood pressure (mm Hg)	—	72.4±14.7	70.7±15.5
Primary Diseases	—	—	—	Diabetes (n = 11); Hypertension (n = 7); interstitial nephritis (n = 3); lupus nephritis (n = 2); IGA nephropathy (n = 1); membranous glomerulopathy (n = 1); cardio-renal syndrome (n = 1); unknown (n = 4)
Medications	—	—	—	ACEi/ARBs (n = 11); Ferrous sulfate (n = 7); Feraheme (n = 1); ESA (n = 7)

### MRI Methods: Acquisition

All MRI data was acquired on a 3.0 T whole body scanner (Magnetom Verio, Siemens Healthcare, Erlangen, Germany). **BOLD MRI** data was acquired in the axial plane using a breath-hold (during end-expiration) multiple gradient echo sequence with the following parameters: FOV = 360 x 245mm, No. of Slice = 5, Slice thickness = 5.0mm, Matrix = 256 x 176, TR = 62ms, No. Echo = 8 equally spaced (3.09–32.3ms), # averages = 1. BOLD MRI measurements were made at baseline and (~15 min) after *iv* administration of 20 mg of furosemide to inhibit sodium reabsorption along the medullary thick ascending limbs which accounts for about 65% of renal O_2_ consumption [13]. **Diffusion weighted imaging** was performed using echo planar imaging (EPI) (Repetition Time (TR)/Echo time (TE)/Flip Angle (FA)/Receiver Bandwidth (BW)/Slice thickness (Thk)/Acquisition Matrix = 3000 ms/78 ms/90°/1630 Hz/vxl/5 mm/192 x 154 and 360 to 420 mm field of view (FOV) with 80% phase FOV). Diffusion sensitizing gradients were applied along three different directions for calculating diffusion trace which is a direction independent measure. Images using four different b values (50, 150, 300 and 1000 s/mm^2^) were acquired and six acquisitions were averaged for improved signal to noise and to minimize motion artifacts. **Renal Volume** was measured using 3D images acquired with a gradient echo sequence (TR/TE/FA/BW/Thk/Matrix/FOV = 3.5 ms/1.6 ms/9° /780 Hz/vxl/3 mm/320x192/380x250 mm) during a breath-hold.

### MRI Methods: Analysis

ADC maps were generated on the scanner platform. For BOLD MRI, the eight images from each mGRE acquisition were used to estimate R2* on a voxel-by-voxel basis, using a linear least squares regression offline using Python 2.7.2. On the magnitude images, a threshold of 20 (a.u.) was used to mask any voxels that decay to the noise floor prior to performing the R2* fit. This was done to minimize the effect of bulk susceptibility artifacts that can distort the estimated value of R2* for each voxel. R2* maps were created for each subject at baseline, and post-furosemide administration.

#### Regions of Interest (ROI)

ROIs were defined in the cortex and medulla for both the left and right kidneys on the first mGRE image. Care was taken not to include any voxels from areas near strong susceptibility changes or other artifacts. Tumors and cysts were excluded from the ROIs as well (3 cases). Medullary ROIs were drawn using a freehand tool and typically consisted of <100 voxels while the cortical ROI was defined as one large ROI (>500 voxels) encompassing the vast majority of the cortex. After all the ROIs were defined for each subject, the mean was computed, resulting in one value per subject per region (cortex and medulla) per stage (baseline and post-furosemide).

For ADC, large regions of interest (ROIs) were selected in the cortex in each of the kidneys and on each slice. Typical ROIs were circular and included at least 100 pixels. Renal volumes were measured by manual tracing of the renal boundary on each slice and then using voxel counting (Image J, NIH) similar to a previous report [14].

### Statistical Analysis

The various MRI derived parameters along with age and eGFR were compared between the CKD and control groups using two-sample T-test. We included Cohen’s *d* value as the measure of the dfference between groups. Cohen’s *d* represents the effect size in three levels: small, medium and large. These levels correspond to *d* values greater than or equal to 0.2, 0.5 and 0.8 respectively [15]. Spearman correlation coefficients were calculated among following variables: R2*_cor, R2*_med, Delta R2*_med (pre R2*_med–post R2*_med), eGFR, ADC and age with *p* value reported. The linear regression was used to explore the pair-wise relationship between R2*_cor, R2*_med, Delta R2*_med, ADC and eGFR. Multiple linear regressions were used to explore the pair-wise relationship accounting for the confounding effect of age and gender where appropriate. Parameter estimate, standard error (SE) and *p* values are reported for the regression analyses.

Aside from the regression analysis, the confounding effect of age and gender in the above-mentioned analysis was removed by conducting a 1:1 age- and gender- matching between CKD and control groups. The propensity score matching method used a greedy 8-to–1 matching algorithm for identifying matched pairs. R2*_cor, R2*_med, Delta R2*_med, eGFR, and ADC were compared between matched CKD and control groups using two-sample T-test. Spearman correlation coefficients were also calculated to see the true relationship among these variables.

All of the statistical analysis were carried out in SAS 9.2 (SAS, Cary, NC, USA), and p<0.05 was regarded as statistically significant.

## Results

### Are there differences between kidneys in healthy vs. subjects with CKD?

Table 2 summarizes the different quantitative parameters measured in the present study. One subject had a recent administration of Feraheme^®^ [S1 Fig] [16] and showed higher renal R2* values (cortex = 44.3 and medulla = 45.8 s^−1^) compared to maximum value observed in the rest of the group. This subject was hence excluded from the rest of the analysis. The table also summarizes the difference in MRI measurements between patients with CKD and controls. The renal volumes are lower and cortical R2* values are higher (suggesting increased hypoxia) in subjects with CKD. Even though medullary R2* did not show a significant increase in CKD, there was a reduced response to furosemide in the medulla. ADC values were not significantly different between the two groups. Since the age between the two groups was significantly different, we further performed comparisons in age and sex matched groups of controls *vs*. CKD (Table 3). Estimated GFR, cortical R2* and kidney volumes remain significantly different while ADC showed no difference. Both medullary R2* and delta_R2* were not different between the groups.

**Table 2:** Summary Table for Variables among groups.

		N	Mean	SD	Median	Min	Max	P value (Healthy *vs*. CKD)	Cohen’s d (Healthy *vs*. CKD)
**Age**	**Anemic**	7	44.48	10.33	42.02	33.65	62.49		
	**Healthy**	22	50.35	15.31	52.12	21.83	76		
	**CKD**	29	61.59	10.42	60.46	37.69	82.25	***0*.*003***	***0*.*86***
**eGFR**	**Anemic**	7	104.86	12.67	105	91	125		
	**Healthy**	22	91.68	12.84	92.78	67	118		
	**CKD**	29	43.25	23.39	34.75	8	93.72	***<* .*0001***	***-2*.*57***
**R**_2_*_cor	**Anemic**	7	18.37	3.02	17.38	16.76	25.19		
	**Healthy**	22	19.68	3.17	18.99	14.54	26.47		
	**CKD**	29	23.2	6.31	20.64	15.49	38.64	***0*.*0126***	***0*.*71***
**R**_2_*_med	**Anemic**	7	31.62	3.71	31.64	24.98	35.22		
	**Healthy**	22	30.11	3.92	29.78	22.09	37.49		
	**CKD**	29	29.15	6.62	28.58	13.52	40.1	0.5206	-0.18
**delta_ R**_2_*_med	**Anemic**	7	9.2	5.92	9.02	-0.35	16		
	**Healthy**	22	6.93	3.31	7.38	1.23	12.93		
	**CKD**	29	3.17	7.49	2.77	-15.81	19.46	***0*.*0205***	***-0*.*65***
**Kidney Volume**	**Anemic**	7	283.17	46.87	285.66	201.96	340.1		
	**Healthy**	19	329.63	66.42	324.31	234.04	453.07		
	**CKD**	23	257.14	86.96	257.53	140.03	448.67	***0*.*0048***	***-0*.*94***
**ADC**	**Anemic**	7	2398.03	400.92	2285.7	1897.77	3180.53		
	**Healthy**	22	1910.42	336.99	1765.16	1564.21	2599.43		
	**CKD**	27	1920.27	374.27	1756.13	1576.24	3070.83	0.912	-0.03

**Table 3:** Summary table for all variables: Age and gender-matched CKD and Controls.

	* *	N	Mean	SD	Median	Min	Max	P value	Cohen’s d
**Age**	**Healthy**	16	57.83	9.21	56	44.39	76		
	**CKD**	16	58	10.57	56.48	37.69	80.17	0.9635	0.02
**eGFR**	**Healthy**	16	88.31	12.33	88.74	67	111.56		
	**CKD**	16	42.24	25.78	34	8	85	***<* .*0001***	***-2*.*28***
**R**_2_*_cor	**Healthy**	16	19.51	3.05	18.93	14.54	26.47		
	**CKD**	16	23.04	5.83	20.96	15.95	38.64	***0*.*0429***	***0*.*76***
**R**_2_*_med	**Healthy**	16	29.85	4.15	29.67	22.09	37.49		
	**CKD**	16	28.97	7.44	27.91	13.52	39.69	0.6835	-0.15
**delta_ R**_2_*_med	**Healthy**	16	6.53	3.24	7.13	1.23	12.93		
	**CKD**	16	2.77	8.37	3.01	-15.81	19.46	0.11	-0.60
**Kidney Volume**	**Healthy**	13	332.28	74.27	371.8	234.04	453.07		
	**CKD**	13	262.36	91.5	257.53	140.03	448.67	***0*.*0428***	***-0*.*84***
**ADC**	**Healthy**	16	1751.28	214.81	1709.88	1564.21	2419.27		
	**CKD**	15	1907.93	317.68	1847.1	1576.24	2477.87	0.1605	0.58

**Fig 1:**
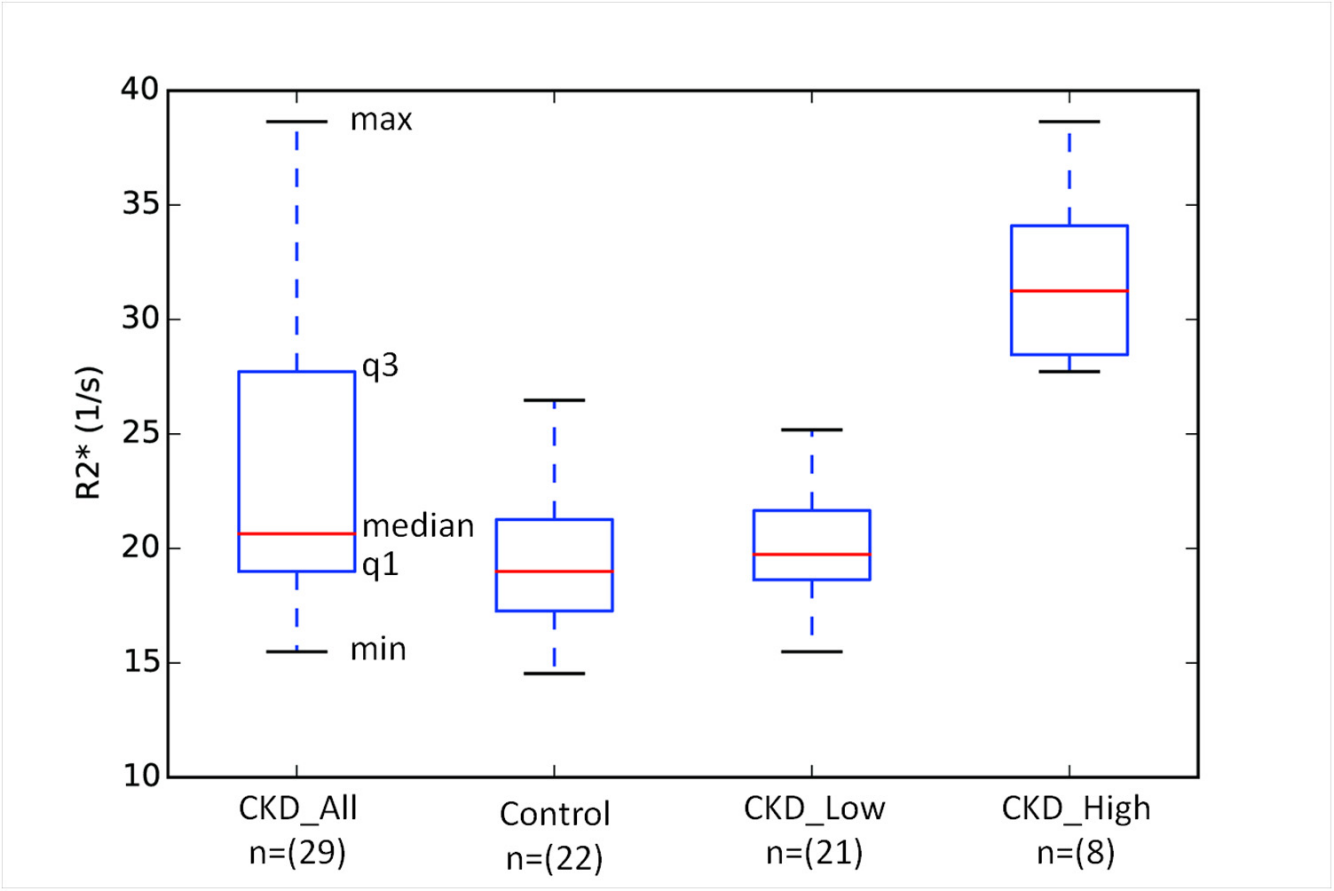
Box-and-whisker plots illustrating the distribution of R2* values in both CKD and control groups. As shown in the annotations, the box-and-whisker plots represent five key values from the distribution (median (red line), 1^st^ quartile (q1), 3^rd^ quartile (q3), minimum and maximum values). For a normal distribution, the median value would be close to the center of the box. The plot for the CKD_All group clearly is skewed with the upper half showing much broader distribution. Also included are distributions separated by a threshold value of 27 s^−1^ (> maximum value in the control group). The CKD_Low group appears to be similar to the control group, while CKD_High is distinctly different.

**Fig 2:**
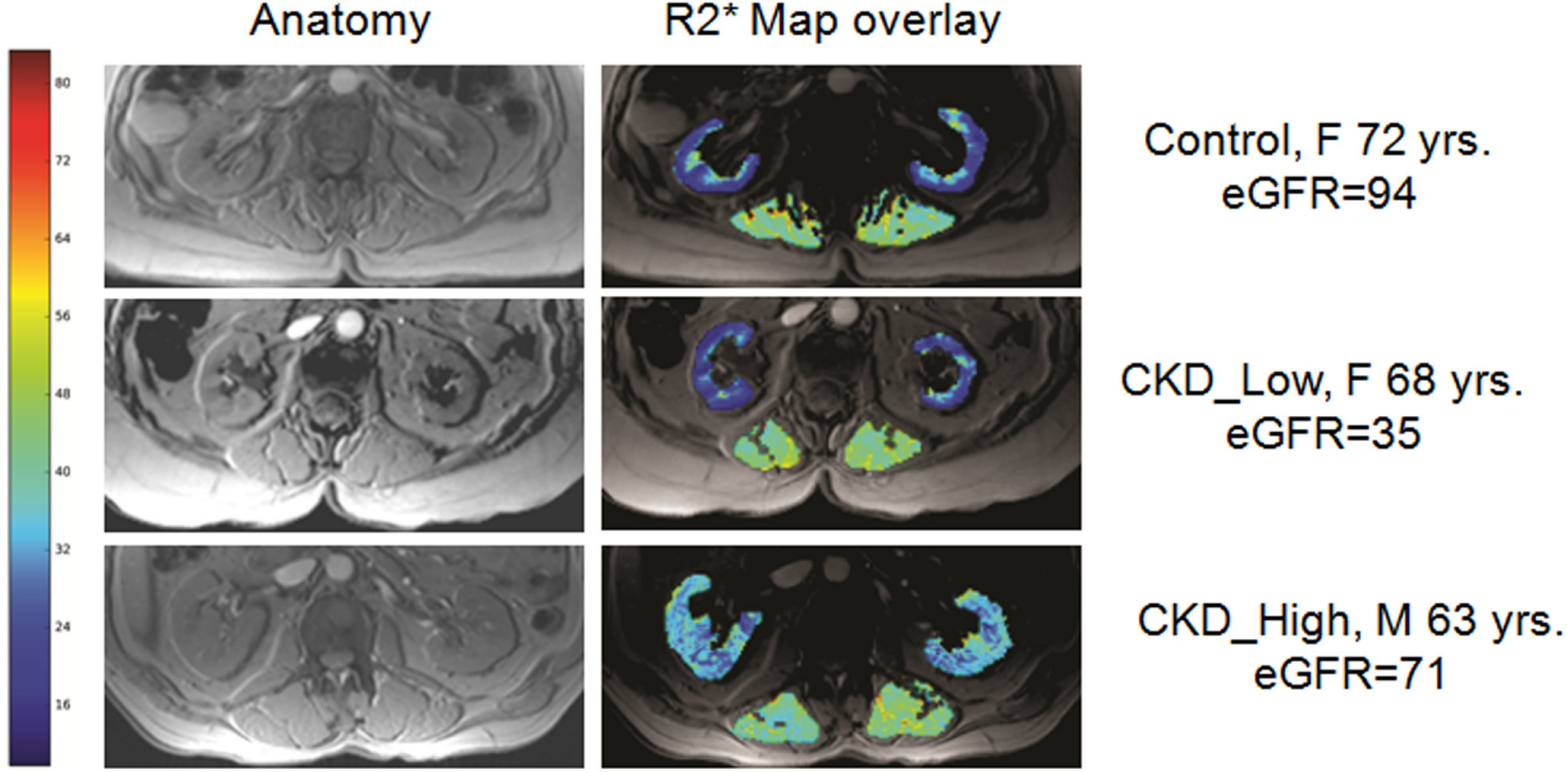
illustrates anatomic MRI and R2* maps from a representative subject from control, CKD_Low and CKD_High R2* groups. The R2* maps of the kidneys are overlaid on the anatomic image and the color bar indicates high levels of hypoxia in red and progressively lower values with orange, yellow, green and blue. Note R2* values are low in the cortex of both the control and subject with CKD even though the eGFR values are significantly different, while the subject with CKD_High clearly shows higher R2* values, even though his/her eGFR values are high and comparable to the control subject. We have also included R2* values for muscle to rule out any systematic bias in R2* values in CKD_High.

Fig 1 summarizes the cortical R2* data in subjects with CKD and healthy controls in the form of box-and-whisker plots. The plot for the CKD_All group suggests that fewer subjects contributed to the higher half of the distribution. Using a threshold of 27s^-1^ (> max value in the control group), we found that the CKD group can be subdivided in to two distinct distributions (CKD_Low (n = 21) and CKD_High (n = 8)) as shown in Fig 1. The CKD_Low group appears very similar to the control group while the CKD_High group has values distinctly higher than either the control or the CKD_Low groups. Fig 2 shows representative images (anatomic and R2* maps) at baseline for each group of subjects for illustrative purposes. Note the similarity of the R2* map in the healthy control and a subject in the CKD_Low group (similar in age and sex) even though the eGFR was substantially different. On the other hand the subject in the CKD_High group with similar age and eGFR as the control subject has much higher cortical R2* values. To rule out any systemic artifactual increase in R2* in the CKD_High group, we have also included ROIs in the muscle, which appears to be comparable in all three subjects. Table 4 summarizes differences between the CKD_Low and CKD_High groups in terms of all the parameters. Except for cortical and medullary R2*, none of the other observed parameters showed significant differences.

**Table 4:** Differences between the subset with High R2* values *vs*. Others among CKD.

	* *	N	Mean	SD	Median	Min	Max	p value	Cohen’s d
**eGFR**	**CKD_High**	8	45.19	22.18	43.00	71.00	10.00		
	**CKD_Low**	21	42.52	24.32	34.00	93.72	8.00	0.78	0.11
**Age**	**CKD_High**	8	61.80	11.86	61.91	82.25	42.58		
	**CKD_Low**	21	61.52	10.13	59.05	81.98	37.69	0.95	0.11
**R**_2_*_cor	**CKD_High**	8	31.99	4.14	31.24	27.72	38.64		
	**CKD_Low**	21	19.85	2.64	19.74	15.49	25.18	***0*.*0001***	***3*.*50***
**R**_2_*_med	**CKD_High**	8	34.18	4.24	33.53	40.10	27.36		
	**CKD_Low**	21	27.23	6.41	27.80	36.58	13.52	***0*.*003***	***1*.*28***
**Delta_R**_2_*_med	**CKD_High**	8	5.31	10.88	6.99	19.46	-15.81		
	**CKD_Low**	21	2.25	5.89	2.34	11.72	-10.45	0.48	-0.34
**Kidney Volume**	**CKD_High**	7	244.46	110.12	192.18	429.65	140.03		
	**CKD_Low**	16	262.68	78.31	262.37	448.67	166.38	0.70	-0.19
**ADC**	**CKD_High**	8	1900.46	491.08	1783.42	3070.83	1576.24		
	**CKD_Low**	21	1928.62	329.09	1756.13	2477.87	1594.50	0.50	-0.07

### Are MRI derived parameters correlated to eGFR?

Table 5 summarizes the pair-wise Spearman correlation analysis performed based on all data shown in Table 2. Estimated GFR and ADC showed a significant dependence on age. Cortical R2*, medullary delta_R2* and kidney volume showed significant dependence on eGFR. A significant correlation was also observed between medullary R2* and delta_R2*. Similarly cortical R2* was significantly and negatively correlated with kidney size. Gender was not found to be a confounding factor for any of the MRI derived parameters. ADC was the only parameter that showed age to be a significant confounder. Table 6 summarizes the linear regression analysis of each parameter with eGFR as the independent variable. Cortical R2*, medullary delta_R2* and kidney volume showed a significant dependence on eGFR while ADC failed to show a similar dependence.

**Table 5:** Pair-wise Spearman correlation among all variables.

* *	age	egfr	R_2_*_cor	R_2_*_med	delta_ R_2_*_med	kidney_volume	ADC
**Age**	1	***-0*.*4955***	0.22668	-0.05875	-0.2023	-0.19909	***-0*.*3327***
**p value**		***<* .*0001***	0.0871	0.6613	0.1277	0.1702	***0*.*0122***
**egfr**		1	***-0*.*3066***	0.16888	***0*.*38367***	***0*.*44391***	0.14758
**p value**			***0*.*0192***	0.205	***0*.*0029***	***0*.*0014***	0.2777
**R**_2_*_cor			1	0.35735	-0.0808	***-0*.*38041***	-0.1911
**p value**				0.0059	0.5465	***0*.*007***	0.1583
**R**_2_*_med				1	***0*.*53717***	-0.07031	-0.1184
**p value**					***<* .*0001***	0.6312	0.3848
**delta_ R**_2_*_med					1	0.03102	0.22509
**p value**						0.8324	0.0953
**Kidney volume**						1	-0.0199
**p value**							0.8944
**ADC**							1

**Table 6:** Linear regression analysis with eGFR as independent variable.

Parameter		Estimate	Standard Error	p value
R2*_cor	**Intercept**	25.34983	1.58721	***<* .*0001***
	**Slope**	-0.06009	0.02112	*0*.*0062*
R2*_med	**Intercept**	27.24934	1.66512	***<* .*0001***
	**Slope**	0.03707	0.02188	0.0958
Delta_R2*_med	**Intercept**	-0.36711	1.82382	0.8412
	**Slope**	0.08241	0.02397	***0*.*0011***
**Kidney Volume**	**Intercept**	204.05373	23.4132	***<* .*0001***
	**Slope**	1.21289	0.30191	***0*.*0002***
**ADC**	**Intercept**	2394.55083	329.393	***<* .*0001***
	**Slope (adjusted for age)**	0.36538	1.85756	0.8448

#### Age and sex matched analysis

Table 7 summarizes the pair-wise Spearman correlation coefficients between the different measurements in the age and sex matched groups. Consistent with Table 5, cortical R2*, medullary delta_R2*, and kidney volume were significantly correlated with eGFR. However, now ADC also exhibited a significant correlation with eGFR. The correlations between cortical R2* and kidney volume, and medullary R2* *vs*. delta_R2* also reached significance.

**Table 7:** Pair-wise Spearman correlation among all variables after matching for age and sex.

	age	egfr	R_2_*_cor	R_2_*_med	delta_ R_2_*_med	Kidney volume	ADC
**Age**	1	-0.0924	-0.0675	-0.14115	-0.1325	-0.10634	-0.1406
**p value**		0.615	0.7137	0.4409	0.4696	0.6051	0.4508
**egfr**		1	***-0*.*3642***	0.16204	***0*.*40235***	***0*.*49889***	***-0*.*391***
**p value**			***0*.*0404***	0.3756	***0*.*0224***	***0*.*0095***	***0*.*0296***
**R**_2_*_cor			1	0.29912	-0.1609	***-0*.*55214***	-0.1637
**p value**				0.0963	0.3789	***0*.*0034***	0.3789
**R**_2_*_med				1	***0*.*51576***	0.00923	-0.3504
**p value**					***0*.*0025***	0.9643	0.0533
**delta_ R**_2_*_med					1	0.22188	-0.2113
**p value**						0.276	0.2539
**Kidney volume**						1	0.03846
**p value**							0.8552
**ADC**							1

When the 8 data points from the CKD_High group were not included, the correlation with eGFR became weak and not significant (ρ = -0.16, p = 0.27).

## Discussion

Our data in a moderately sized sample shows significant differences between kidneys of subjects with CKD compared to healthy controls. Cortical R2* and kidney volume showed significant differences between the groups with or without age and sex matching. While medullary delta_R2* (response to furosemide) was significantly different when using the entire group, the difference did not reach significance when using age and sex matched samples, even though similar trend of reduced response in CKD was observed. The absence of a significant response to furosemide in the age matched group may suggest age dependence of the response in healthy subjects as indicated by a prior report [17]. Cortical R2*, kidney volume and medullary delta_R2* showed a significant correlation with eGFR with and without age and sex matching. These observations collectively suggest that cortical R2*, kidney volume and medullary delta_R2* can be useful in the evaluation of subjects with CKD. The relationship of renal size with renal function is known [12]. However, most data to-date is based on ultrasound. MRI based voxel counting method has been shown to be more accurate for volume measurements compared to ellipsoid formula used for ultrasound measurements [14]. The difference in medullary response to furosemide between controls and subjects with CKD is consistent with a recent report [18]. While the differences in cortical R2* are predicted by the chronic hypoxia theory, recent reports have been controversial [19–24].

The study by Inoue *et al* [19] was performed in a sufficiently large number of subjects (n = 142) and reported significant increase in cortical R2* between subjects with CKD compared to healthy controls. Our observations are consistent with these findings. A subsequent study by Long *et al* [23] was performed in a smaller number of subjects (n = 15), primarily patients with very early CKD (GFR > 60 ml/min/1.73 m2). They found the medullary R2* values were significantly higher in the patients with CKD. Yin *et al* [24] reported increased levels of cortical R2* and decreasing levels of medullary R2* with lower eGFR. Wang *et al* [22] reported decreased medullary R2* values with no change in cortical R2* compared to controls. Three of the four studies involved restriction of fluid intake, similar to the present study. Inoue *et al* [19] did not specify any fluid restriction in their subjects, but do believe variation in hydration status mostly affect medullary oxygenation and hence only reported cortical measurements. Michaely *et al* [20] reported on BOLD MRI in relation to eGFR in a study with a large number of subjects (n = 400). The study was performed in subjects undergoing abdominal MRI scans for various reasons. BOLD MRI data was acquired additionally and in a subset of subjects (n = 280) where serum creatinine data was available, a relationship between R2* and eGFR was evaluated. They reported that neither cortical nor medullary R2* was correlated to eGFR and hence concluded that BOLD MRI is not sensitive to CKD which led to several rebuttal discussions [25–27]. The study did not include any subject preparation nor a control group. A subsequent study by Pruijm *et al* [18] did include subject preparation, but also reported no differences in R2* measurements between CKD (n = 95) and healthy controls (n = 42). However in a subsequent report, when using a more objective analysis of R2* maps, the authors did observe an increased cortical R2* in subjects with CKD [28] similar to the present study. The use of large cortical ROIs in this study has been shown to be objective [29] and is similar to the outer layer(s) in Pruijm’s analysis [28].

While our data showed statistically significant differences in cortical R2* between subjects with CKD and controls, as seen in Fig 1 there is a large overlap in the distribution of values between the two groups. The differences were observed even when matching for age and sex between the groups [Table 3]. While this is of some limited academic interest, it will have minimal impact in practice. For translation to the clinic, where decisions will be made on an individual basis, overlap should be minimal to none. This led us to further evaluate the observed distribution of cortical R2* values in the CKD group which clearly is skewed, suggesting that a few data points with high R2* values were contributing to the observed mean value. Based on the fact that there were data points beyond the maximum value in the control group, we used the maximum R2* value in the control group (26.7 s^−1^) as a threshold and sub-divided the CKD_All group in to CKD_Low and CKD_High groups. As seen from Fig 1, these two groups are distinctly different (*i*.*e*. with no overlap), and the distribution of CKD_Low was similar to the control group.

Based on the above observation, we believe the reason for the apparent discrepancy between recent reports could be related to the fact that a majority of the subjects with CKD may not show a difference compared to healthy controls on BOLD MRI, with only a minority (possibly the ones at risk of progression) showing large differences. When the 8 data points from the CKD_High group were not included, the correlation with eGFR became weak and statistically insignificant. These observations are consistent with the recent realization that progression in CKD follows a non-linear trajectory with long periods of stable renal function [30]. It is also interesting that the ratio of 8/29 is comparable to 31/96 reported in a study using neutrophil gelatinase-associated lipocalin (NGAL) as a predictive marker for progression in CKD [31]. Because most of the current studies are based on volunteer participation, they are not necessarily true random statistical samples of the general CKD population. It is conceivable that certain studies may be more weighted by non-progressors and so the subjects appear not to be different from controls.

Interestingly, the majority of the subjects (6 out of 8) in the CKD_High group were CKD stage 2 (n = 3) or 3 (n = 3). One was CKD–4 and another CKD–5 and these two were associated with the two highest R2* values recorded. In terms of primary disease there was one each with diabetes, hypertension, lupus, IGA nephropathy and membranous glomerulopathy. The other three were unknown. Two out of the 8 subjects were taking ferrous sulfate and two others were taking ACEi/ARBs. One was taking both. These observations may suggest that even mild to moderate CKD independent of etiology can be associated with high R2* values. However, due to the lack of another independent marker for progression we are not able to associate these observations with progression. These data however strongly support the need for longitudinal studies to determine whether such association exists.

There were a few secondary observations of interest. Since BOLD MRI is inherently dependent on the presence of hemoglobin in blood, the R2* measurement is dependent on the hematocrit. The effect of reduced hematocrit is to reduce R2* values (due to lower susceptibility effects) as demonstrated in a previous report in rat brains where hemodilution was used to vary hematrocrit [32]. A small group of anemic subjects due to non-renal etiology were included in this study to allow us to estimate the potential contribution of reduced hematocrit on renal BOLD MRI. Our data did show slightly lower cortical R2* values in anemic compared to healthy subjects (18.4±3.0 *vs*. 19.7±3.2 s^−1^), even though the differences did not reach statistical significance. The smaller change in our study compared to the hemodilution maneuver in animals [32] may be related to the differences in the magnitude of change in hematocrit. Since the hemoglobin levels in the CKD group was actually slightly higher compared to the anemic subjects, we could assume the contribution from the reduced hematocrit to be even smaller. It is also important to note that the effect of lower hematocrit on R2* is opposite in nature compared to lower oxygenation which increases R2*. So, the levels of hypoxia observed in the kidneys may actually be an underestimate due to the associated anemia in subjects with CKD. Overall, for practical purposes, we can assume the contribution to the observed renal R2* values from anemia associated with CKD may be small.

The reduced response to furosemide is consistent with another recent report [18]. An exact explanation for the reduced response is not yet clear. It may simply be a dose effect, *i*.*e*. the dose of furosemide in CKD needs to be higher to elicit a similar response in people with normal GFR. The fact that delta_R2*_med was significantly correlated with eGFR supports such a dose effect. Alternately, it is possible that the medullary blood flow reductions associated with furosemide [33] may reduce the decrease in R2* post-furosemide. Whether change in medullary blood flow following furosemide is different in CKD is not yet known. With the lack of a non-invasive method to measure medullary blood flow *in vivo* in humans, this may be difficult to verify.

Subjects who were taking angiotensin converting enzyme inhibitors (ACEi) or angiotensin receptor blockers (ARB) demonstrated no statistical significant differences in either cortical (24.1±6.3 (n = 11) vs. 22.84±6.7 (n = 15), p = 0.62) or medullary R2* values (30.0±4.7 (n = 11) vs. 28.3±8.24 (n = 15), p = 0.5) compared to those who were not. While it has been shown that ACEi can reduce renal R2* values on an individual basis when administered acutely [34] or chronically [35], the effect may be small such that it does not affect group differences. Alternately, it could imply that at the ACEi dose and frequency of dosing currently used, may not be efficacious at improving renal hypoxia on a chronic basis.

ADC failed to show differences between controls and subjects with CKD. There was a significant correlation observed with age. When matched for age and sex, ADC had a significant correlation with eGFR. Few prior studies have shown renal ADC to be different between subjects with CKD and healthy controls [19,36–38]. However all these studies included b = 0 in their analysis. Three of these studies only used two b values [36–38]. It could be argued that inclusion of b = 0 may include effects of other compartments with high ADC values such as the blood and tubular compartments. We used b values above 50 mm^2^/s in order to avoid the effects of blood and tubular compartments. Our primary interest was to evaluate whether ADC measurements are sensitive to the presence of fibrosis which primarily affects the tissue compartment.

Our study did have some limitations. Primarily the number of subjects with CKD was small especially for advanced stages. This is more relevant due to the multi-factorial nature of CKD. We did not have histological confirmation for the presence of fibrosis and/or hypoxia. The smaller number did not permit analysis for separating diabetics and other etiologies as performed by Inoue *et al* [19]. Volume measurements could benefit by correcting for subject height in terms of improved correlation with eGFR [12]. The limitation of using blood oxygenation as a surrogate for tissue oxygenation may fail at advanced stages due to the loss of vasculature and red blood cell mass [25]. The inherent dependence of R2* on R2 makes it potentially sensitive to changes in blood or tubular volume [39]. Based on a recent report [11], the b values used for ADC measurements should be > 300 s/mm^2^ for the purpose of evaluating tissue fibrosis. Alternately, additional b values can be included and a bi-exponential fitting can be used to evaluate both fast and slow diffusion compartments [40]. Future studies should consider alternate dosing of furosemide, perhaps normalized to body weight.

## Conclusions

These data suggest increased renal hypoxia can be detected by BOLD MRI in subjects with CKD. Our results do not support the use of diffusion MRI as a marker for differentiating kidneys in subjects with CKD from healthy controls. Renal parenchymal volume was significantly smaller in subjects with CKD. Renal volume, cortical R2* and medullary response to furosemide showed a significant correlation with eGFR. Even in the age and sex matched sub-group, these correlations remained significant. In this regard, it is important to note that a majority of the subjects with CKD (irrespective of their stage) had comparable cortical R2* values to the control group. Only a small number of subjects with varying stages of CKD had much higher values of R2* contributing to the higher group mean observed. The effect size (as estimated by Cohen’s d) for the difference between the subjects in the CKD_High and CKD_Low groups was 3.5, which suggests very high sensitivity. These observations are consistent with the chronic hypoxia model proposed by Fine and Norman suggesting the role of hypoxia in the development of progressive CKD [7]. Further studies are warranted to demonstrate whether the increase in R2* (and hence hypoxia) is associated with risk of progression. Once demonstrated, BOLD MRI could be utilized to determine the presence of hypoxia in patients with early stages of CKD and stratify which patients are at risk of progressive disease. More aggressive management could then be focused on those subjects who would likely have greater benefit and decrease the risks and costs of aggressive management to those patients who would otherwise not benefit from such treatments. Future studies could also include cortical perfusion measurements using arterial spin labeling that has shown reduced cortical perfusion in patients with CKD [41].

## Supporting Information

S1 Dataset This file contains raw data necessary to reproduce our results. (PDF)

S1 Fig The first image of the 8 gradient echo images acquired for R2* mapping from the subject who received Feraheme^®^ recently for treating anemia. Note the dark liver (arrow) which is known to persist for few weeks following administration [16]. A representative example from another subject is shown for reference. The exact effect on renal R2* due to persisting Feraheme^®^ is not yet known.
(TIF)
