# Supplementary material for: Multi-Parametric Evaluation of Chronic Kidney Disease by MRI: A Preliminary Cross-Sectional Study
Source: PLoS One. 2015 Oct 2;10(10):e0139661. doi: 10.1371/journal.pone.0139661 (PMC4591972; doi:10.1371/journal.pone.0139661)
Supplement: S1 Dataset — (PDF) [file pone.0139661.s001.pdf]

Age at Exam	Study Code	Male/Female	Subject Type	eGFR	R2*_Cortex	R2*_Med	Delta R2* Med	L+R Volume	ADC_cortex
33.7	NK21	F	Anemic	96	17.24	34.23	15.63	201.96	2255.60
41.0	PK23	F	Anemic	111	17.47	35.19	11.55	291.88	2263.34
34.4	LS24	F	Anemic	114	17.55	24.98	4.19	253.41	2285.70
62.5	EL25	F	Anemic	125	17.38	31.64	8.38	331.62	3180.53
53.4	MS28	F	Anemic	105	17.00	30.67	16.00	277.54	2301.53
44.4	KD30	F	Anemic	91	16.76	29.39	9.02	340.10	2601.77
42.0	JK47	F	Anemic	92	25.19	35.22	-0.35	285.66	1897.77
21.8	MJ01	M	Healthy	94	16.41	28.32	1.99	300.99	2156.13
29.7	RA03	M	Healthy	88	18.84	31.99	10.82	285.93	2527.80
28.9	AO04	M	Healthy	100	19.32	26.83	7.08	324.31	2114.00
30.0	AT07	M	Healthy	100	17.03	36.28	12.08	346.41	2212.77
44.4	DD08	M	Healthy	81	16.00	28.62	12.93	376.39	2419.27
46.7	GP09	M	Healthy	104	26.16	32.49	7.21	273.07	2599.43
25.3	JK14	M	Healthy	118	22.97	28.85	8.84	412.58	2398.57
62.0	SA40	M	Healthy	86	20.00	22.09	3.05	386.38	1763.79
51.2	BJ42	M	Healthy	79	22.01	37.49	10.92	453.07	1911.53
47.7	TF43	F	Healthy	67	26.47	30.71	3.01	242.34	1977.40
62	CF505	F	Healthy	93.75	20.43	33.08	6.70	283.64	1595.98
53	DF509	M	Healthy	92.41	18.58	28.61	2.40	292.25	1647.02
55	DH507	M	Healthy	111.56	14.54	27.90	8.14	405.88	1741.52
57	FW215	F	Healthy	77.15	16.95	28.31	5.13		1710.37
73	MA502	F	Healthy	89.45	24.09	26.11	5.28	234.04	1709.39
51	MB206	F	Healthy	88.02	18.71	33.39	4.31		1807.59
72	MH501	F	Healthy	94.86	20.97	32.32	8.70	250.43	1564.21
58	PK503	M	Healthy	104.64	17.99	31.39	7.55	392.16	1587.17
53	PP506	M	Healthy	103.06	21.36	33.39	8.62	255.63	1583.56
59	SS508	M	Healthy	93.16	16.72	33.81	8.60	371.80	1642.71
51	TF220	F	Healthy	68.48	19.14	22.64	1.23		1766.52
76	TT504	M	Healthy	83.44	18.22	27.78	7.88	375.63	1592.51
73.1	JK05	F	Patient	8	19.61	16.26	2.25	166.38	2477.87
68.2	JS10	F	Patient	28	22.34	33.97	-0.89	197.67	1928.53
56.4	AB11	M	Patient	64	19.74	20.16	-5.48	448.67	2023.30
68.0	ML13	M	Patient	32	19.99	31.68	8.40	267.21	2434.33
60.5	NS15	F	Patient	38	20.64	20.86	0.47	204.41	2314.97

59.0	PJ16	M	Patient	16	18.64	27.26	9.95	220.97	2276.87	
60.7	KT20	F	Patient	46	28.54	35.22	9.31	152.90	3070.83	
37.7	JH26	F	Patient	85	17.86	28.02	9.20	329.63	0.00	
80.2	SM31	M	Patient	17	15.95	13.52	-6.00	370.79	2280.21	
55.9	AC32	F	Patient	65	15.49	24.98	2.34	275.74	1632.23	
57.5	RN33	M	Patient	52	17.17	28.40	7.09	330.11	1756.13	
52.3	SY34	F	Patient	34	21.66	35.86	11.72	257.53	1628.95	
53.2	AW35	F	Patient	34	25.18	27.80	3.24	173.01	1627.13	
52.8	AJ36	F	Patient	71	20.27	26.92	-3.90	284.34	2439.93	
42.6	RE38	F	Patient	10	38.64	27.36	-15.81	140.03	1599.97	
82.0	LG39	M	Patient	23	21.87	28.58	1.68	283.77	1638.47	
55.9	NO41	F	Patient	33	15.69	19.48	-10.45	201.90	0.00	
55.4	GF44	M	Patient	69	30.55	31.75	4.67	306.66	1847.10	
65.2	DF45	M	Patient	30	28.23	34.67	-0.58	324.19	1576.24	
71.0	SC46	M	Patient	40	31.93	32.39	11.78	192.18	1929.53	
64.3	MN48	M	Patient	18	23.49	36.58	7.83	190.79	1681.60	
63.1	CB49	M	Patient	71	33.02	32.27	0.31	429.65	1610.70	
82.2	LR50	F	Patient	29	37.31	40.10	13.33	165.65	1719.74	
66.2	CK51	F	Patient	21.03	18.76	24.57	-0.10		1730.00	
68.4	SD52	F	Patient	34.75	19.71	31.08	-2.95		1594.50	
67.1	RS53	M	Patient	93.72	24.05	32.07	6.30		1796.96	
56.8	JP54	M	Patient	69.43	18.99	36.25	5.92		1655.73	
56.6	DC55	M	Patient	55.92	19.77	27.51	2.77		1725.97	
54.2	MB56	M	Patient	66.48	27.72	39.69	19.46		1849.57	
64.2	NG18	F	Patient	9	44.30	40.58265929	5.22	169.43	2092.40	Not used in analysis
