# Supplementary figures and images for: Multi-Parametric Evaluation of Chronic Kidney Disease by MRI: A Preliminary Cross-Sectional Study
Source: PLoS One. 2015 Oct 2;10(10):e0139661. doi: 10.1371/journal.pone.0139661 (PMC4591972; doi:10.1371/journal.pone.0139661)

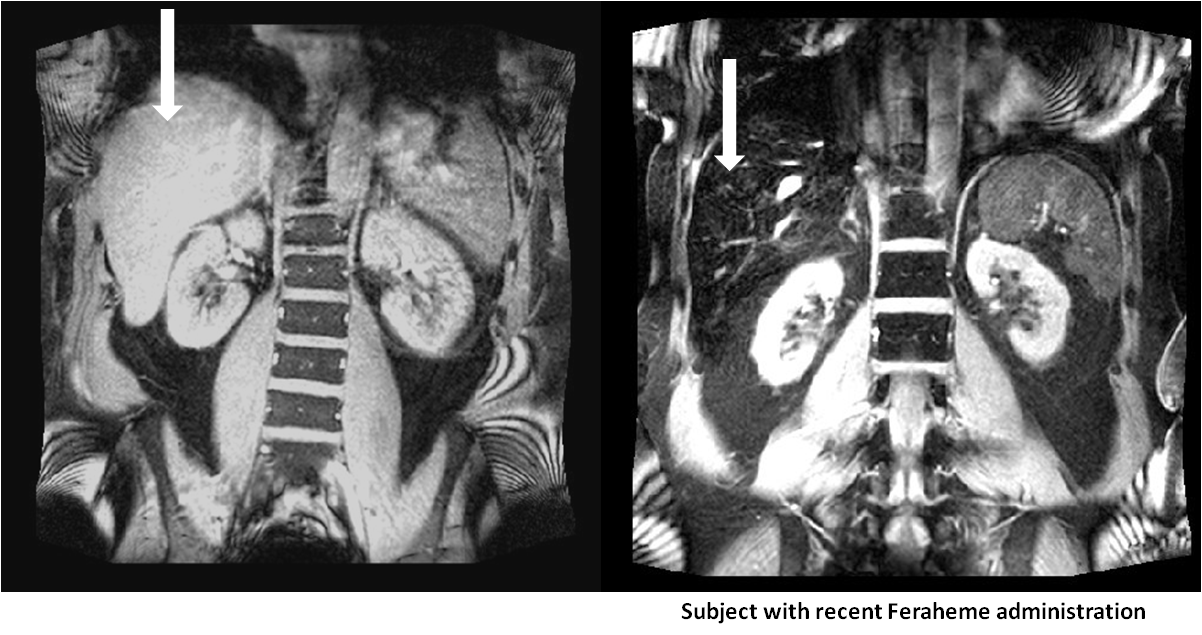

Supplement: S1 Fig — Note the dark liver (arrow) which is known to persist for few weeks following administration [16]. A representative example from another subject is shown for reference. The exact effect on renal R2* due to persisting Feraheme® is not yet known. (TIF) [file pone.0139661.s002.tif]
